# MicroRNAs in the regulation of Th17/Treg homeostasis and their potential role in uveitis

**DOI:** 10.3389/fgene.2022.848985

**Published:** 2022-09-14

**Authors:** Fen Tang, Zhou Zhou, Kongqian Huang, Wen Deng, Jiali Lin, Ruoyun Chen, Min Li, Fan Xu

**Affiliations:** Department Of Ophthalmology, The People’s Hospital Of Guangxi Zhuang Autonomous Region and Institute Of Ophthalmic Diseases, Guangxi Academy Of Medical Sciences, Nanning, Guangxi, China

**Keywords:** microRNAs, autoimmunity, Treg cells, Th17 cells, intermediate, posterior, uveitis (MeSH)

## Abstract

Th17 and regulatory T cells (Tregs) play crucial roles in the pathogenesis of autoimmune diseases. Th17/Treg homeostasis is critically involved in maintaining the immune balance. Disturbed Th17/Treg homeostasis contributes to the progression of autoimmune diseases. MicroRNAs (miRNAs) have emerged as a new vital factor in the regulation of disturbed Th17/Treg homeostasis. To better understand the epigenetic mechanisms of miRNAs in regulating Treg/Th17 homeostasis, we included and evaluated 97 articles about autoimmune diseases and found that miRNAs were involved in the regulation of Treg/Th17 homeostasis from several aspects positively or negatively, including Treg differentiation and development, Treg induction, Treg stability, Th17 differentiation, and Treg function. Uveitis is one of the ocular autoimmune diseases, which is also characterized with Th17/Treg imbalance. However, our understanding of the miRNAs in the pathogenesis of uveitis is elusive and not well-studied. In this review, we further summarized miRNAs found to be involved in autoimmune uveitis and their potential role in the regulation of Th17/Treg homeostasis.

## Introduction

### Disturbed Th17/Treg homeostasis in autoimmune diseases and uveitis

Multiple studies have demonstrated that disturbed Th17/Treg homeostasis plays a pivotal role in the progression of many autoimmune diseases, such as rheumatoid arthritis (RA) (Wang et al., 2012), systemic lupus erythematosus (SLE) (Alunno et al., 2012), and autoimmune uveitis. Th17 cells are defined as effector T lymphocytes (Teffs) that contribute to inflammation development. They could produce pro-inflammatory cytokines, such as interleukin (IL)-17A, IL-17F, IL-21, IL-22, and TNF-a, which are pathogenic in various autoimmune diseases (Sutton et al., 2009). Overall, the high frequency and elevated function of Th17 can induce inflammation. Treg cells (Tregs) are CD4^+^CD25 + cells that express the transcription factor fork-head box P3 (FoxP3), which controls critical transcriptional programs for Treg cells’ function. They could regulate immune responses and maintain self-tolerance (Wan and Flavell, 2008; Fujio et al., 2010). The loss of Tregs or their function results in fatal autoimmune diseases in mice and humans (Sakaguchi et al., 2020). To summarize, Th17 cells contribute to active autoimmunity and autoimmune disease progression by secreting IL-17A and other pro-inflammatory cytokines, while Tregs control active autoimmunity and establish self-tolerance by inhibiting effector T-lymphocyte proliferation and suppressing their function (Naqvi et al., 2021; Zhou et al., 2007).

There is a consensus of opinion that the disruption of the balance between pathogenic Th17 and Tregs would result in the breakdown of self-tolerance and contribute to autoimmune disease development (Paust and Cantor, 2005; Tang and Bluestone, 2006). Similarly, maintaining Th17/Treg balance is considered a key factor for the treatment strategy of autoimmune diseases, including uveitis. Autoimmune uveitis is also a CD4^+^ T cell-mediated autoimmune disease (Zhou et al., 2007). It has been demonstrated that decreased frequency and diminished function of Treg cells would contribute to the inflammation progress in an animal model of experimental autoimmune uveitis (EAU). Moreover, the inflammation was significantly alleviated by shifting the imbalance with induced Tregs. Furthermore, it also had been verified that the disturbed Th17/Treg balance was closely associated with active uveitis such as Vogt-Koyanagi-Harada disease (VKH) and Behcet’s disease (BD) (Chen et al., 2008; Nanke et al., 2008). However, the regulation mechanism of the disturbed Treg/Th17 balance is still unclear.

### MicroRNAs are critically involved in the regulation of Th17/Treg homeostasis

Recently, microRNAs (miRNAs) have emerged as critical regulators in Th17/Treg homeostasis. miRNAs are a novel group of small ncRNA molecules of 19–24 nucleotides (nt) in length that participate in the post-transcriptional regulation of gene expression mostly by pairing with 3′UTR of their mRNA targets and inhibition of its translation (Sato et a., 2011). Both innate and adaptive immunities are highly regulated at the post-transcriptional level with miRNA interference (Tsitsiou and Lindsay, 2009; Wei et al., 2020). Multiple studies showed that miRNAs are pivotal regulators involved in autoimmune diseases and can be used as an epigenetic regulation in immune response.

Studies showed that the miRNA network had a profound role within T-cell biology, including T-cell differentiation, T-cell proliferation, cytokine secretion, and Treg cell function. To gain functional evidence of the role of miRNAs in Treg/Teff biology, researchers have used the CD4-Cre Dicer deletion mouse models to analyze the development and function of T cells. Cobb et al. (2006) indicated that depleting some miRNAs by eliminating Dicer reduced the number of peripheral Treg cells. Chong et al. (2008) found that the lack of miRNAs resulted in a two- to three-fold decrease in the frequency of Tregs (Zhou et al., 2008). In addition, some studies with animal models reported that some miRNAs were involved in the progression of autoimmune diseases by regulating Foxp3 in Treg cells (Liston et al., 2008; Zheng et al., 2007). Overall, these studies have clearly demonstrated that the immunosuppressive mechanism in Treg cells was controlled by miRNAs. However, although this critical role has been firmly demonstrated, key mechanisms of the miRNA function in regulating Treg cell development and function remain elusive.

### The potential role of miRNAs in the pathogenesis of uveitis

Autoimmune uveitis is an intraocular autoimmune eye disorder which is characterized with immune-mediated damage in the uveal, vascular, and retina tissues. The progressive damage of photoreceptors caused by autoreactive T cells eventually leads to irreversible visual impairment and even blindness (Caspi, 2010). It was estimated that it accounts for 10%–25% of blindness globally (Gritz and Wong, 2004). Although its pathogenesis is comprehensive and still not clear, immunological abnormality, especially Th17/Treg homeostasis, is widely considered as the pivotal factor for its etiology. Similarly, miRNAs have also been investigated for their roles in uveitis pathobiology as biomarkers or therapeutic targets. However, the detailed mechanisms of miRNAs for the abnormal immune system in uveitis still need to be further explored.

In the review, the miRNAs involved in Th17/Treg homeostasis and their regulatory network have been summarized first. We aimed to integrate the studies on individual miRNAs into a more global understanding of the function of the miRNAs in the regulatory network of Treg/Th17 balance. Moreover, considering the critical role of disturbed Th17/Treg homeostasis in uveitis pathology, we then reviewed the possible miRNAs in uveitis physiology by regulating Treg/Th17 hemostasis, aiming to improve the mechanistic understanding of miRNA biology in uveitis.

## Methods

### Search strategy and literature search

**FIGURE 1:**
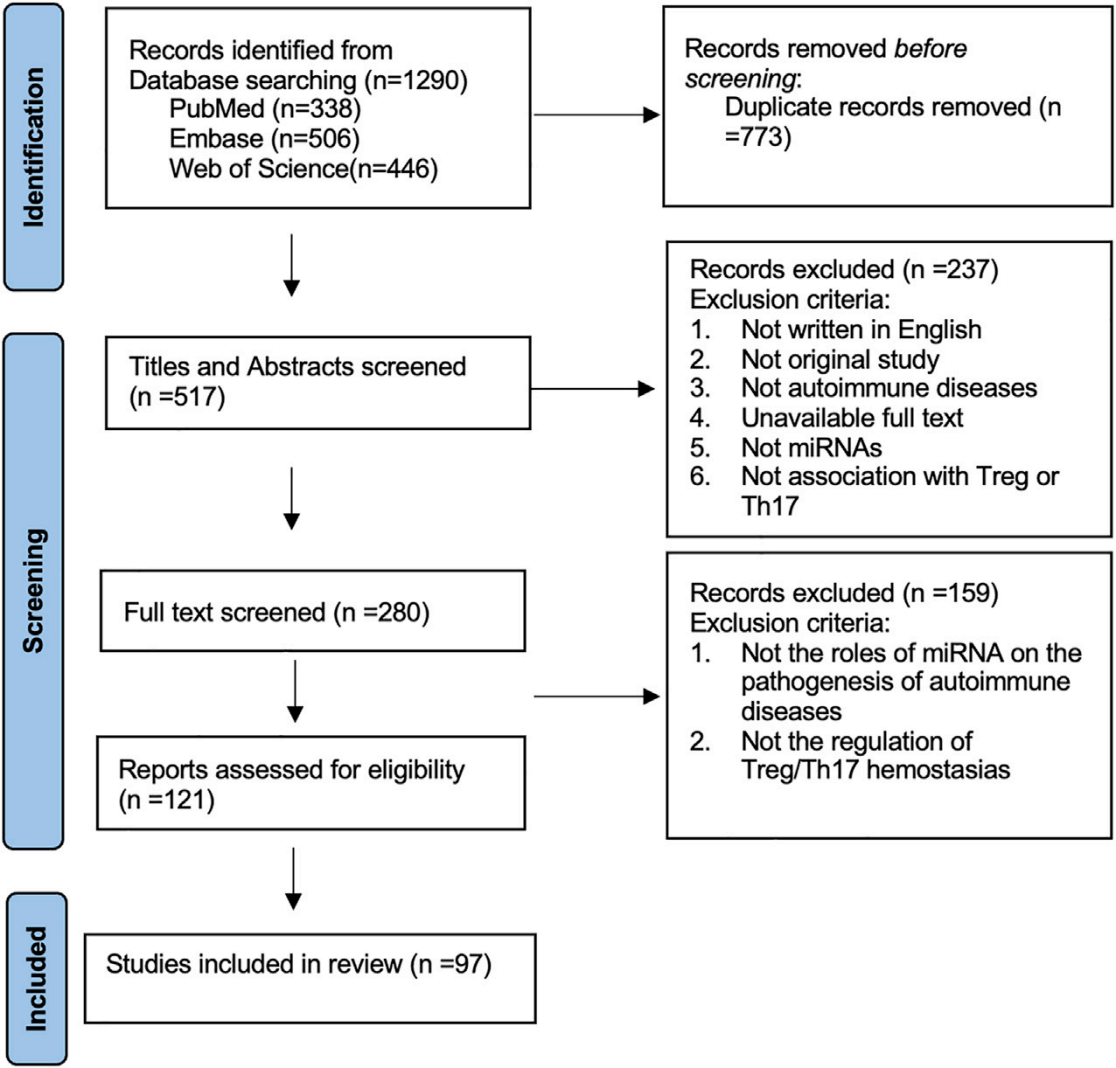
Flow diagram for systematic review.

This study was conducted according to the Preferred Reporting Items for Systematic Reviews statement. We conducted a search of journal articles from several databases published from 2010 until the present day, including PubMed, Web of Science, and Embase. The terms chosen were autoimmune inflammation OR autoimmune disease and microRNA* OR miRNA* OR miR* and Th17 OR Treg. Initially, 1,290 articles were recovered. Then, duplications of articles were removed, and after removing 773 duplicates, the articles’ abstract and title were assessed subsequently. In total, 280 articles were subjected to full-text screening. Based on full-text screening and eligibility assessment, 97 articles were included into the systematic review eventually. The roles of miRNAs in the pathogenesis of autoimmune diseases by regulating Th17/Treg homeostasis were reviewed and summarized. The exclusion criteria are shown in the PRISMA flow diagram. The systematic review process is shown in Figure 1.

Moreover, we searched human or animal studies about the miRNAs in the pathogenesis of uveitis by regulating Th17 or Treg cells. Terms chosen were uveitis and microRNA* OR miRNA* OR miR* and Th17 OR Treg. The exclusion criteria were as follows: 1) Full text unavailable; 2) not written in English. Finally, eight animal studies about experimental autoimmune uveitis (EAU) and nine human studies about Behçet’s disease and Vogt-Koyanagi-Harada (VKH) syndrome were included for further review. The following data were extracted: first name of the author, year of publication, study species, miRNA, and the potential role of miRNA in the Th17/Treg homeostasis.

## Results

### miRNAs involved in Th17/Treg homeostasis by regulating Treg differentiation and development

**FIGURE 2:**
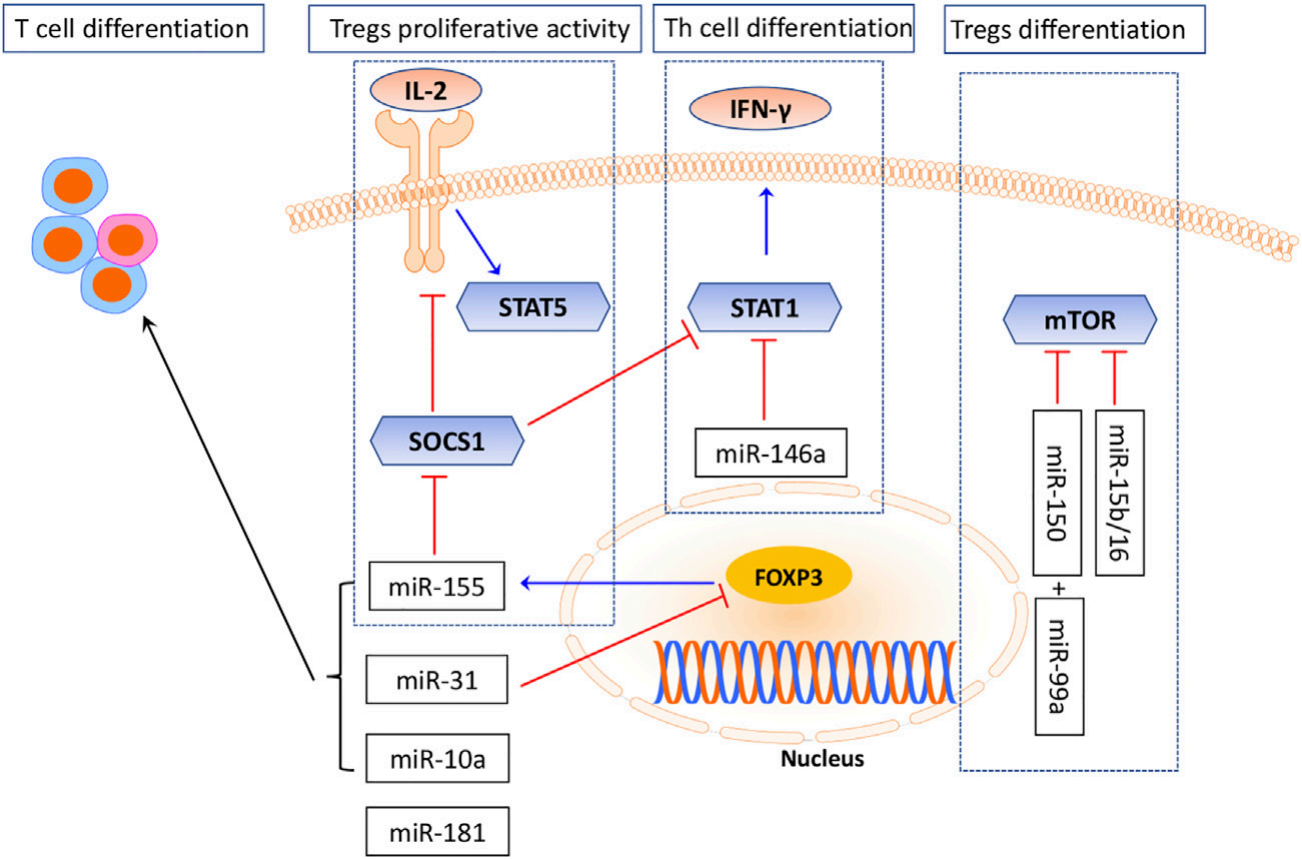
miRNAs involved in Treg differentiation and development.

Several miRNAs had been demonstrated to regulate Treg differentiation and development, particularly miR-155 and miR-146a (Rodriguez et al., 2007). miR-155 is processed from an exon of non-coding RNA. Its expression was greatly upregulated in active T cells (Eis et al., 2005; van den Berg et al., 2003). The critical role of miR-155 in the differentiation of T cells and its subtype Tregs was obtained from studies in miR-155-knockout mice, which failed to develop a protective response after immunization (Thai et al., 2007). The miR-155-knockout mice were found to display a bias toward T helper 2 (Th2) differentiation, indicating that miR-155 promotes differentiation into Th1 cells (Naqvi et al., 2021). Furthermore, miR-155 was also involved in regulating Treg cell differentiation, maintenance, and function by targeting FoxP3 (Lu et al., 2009; Mahesh and Biswas, 2019), which binds to an intron within the DNA sequence encoding the miR-155 precursor mRNA and thereby maintaining the high levels of miR-155 expression in Tregs (Heyn et al., 2016; Hippen et al., 2018). The number of Tregs in the thymus and periphery is decreased significantly if miR-155 is downregulated (Kohlhaas et al., 2009). Moreover, Lu et al. (2009) demonstrated that Foxp3-dependent miR-155 sustained IL-2R signaling by targeting the suppressor of cytokine signaling 1 (SOCS1) protein. In the absence of miR-155, increased amounts of SOCS1 attenuate the IL-2R pathway, leading to reduced activating transcription factor signal transducer and the activator of transcription 5 (STAT5) phosphorylation and reduced competitive fitness. Conclusively, as is shown in Figure 2, these studies suggested a positive role of miR-155 in sustaining Treg proliferative activity and numbers *via* its inhibition on SOCS1, a negative regulator of the IL-2 pathway (Yao et al., 2012; Yin et al., 2018). However, several studies have demonstrated that SOCS1 is necessary for the functions of Tregs and SOCS1 knockout in Tregs would lose Foxp3 expression, which seems controversial with the role of miR-155. Nevertheless, like a double-edged sword, SOCS1 may have negative or positive effects under different circumstances.

Another key miRNA is miR-146a. Its deficiency would result in an increased number of Treg cells and could cause a defect in their immunosuppressive response (Holmstrom et al., 2017; Tang et al., 2009).miR-146a was mainly expressed in Treg cells and affected Treg cell’s ability to suppress the Th1 response by targeting the signal transducer and activator of transcription 1(STAT1), which is an important factor for Th1 differentiation (Lu et al., 2010). STAT1 is a key transcription factor in the IFN-γ response (Lu et al., 2010). In addition, SOCS1 is also a negative regulator of STAT1; STAT1 activation with ablation of SOCS1 resulted in a similar Th1-mediated pathology and differentiation (Lu et al., 2009; Lu et al., 2010).

Except that, there are some other miRNAs which are critically involved in Treg differentiation and development. As is shown in Figure 2, miR-31 was shown to inhibit T-cell differentiation into Tregs by binding to a potential target site within 3’ UTR of FoxP3 mRNA and downregulating its synthesis (Allantaz et al., 2012). miR-10a negatively regulates the plasticity of peripheral Tregs by differentiating them into T follicular helper (Tfh) cells (Sethi et al., 2013; Wu et al., 2019). mTOR exerts a critical role in inhibiting Treg differentiation *via* miRNA mediation (Delgoffe et al., 2009; Warth et al., 2015). Studies showed that miR-99 and miR-150 work in concert to repress mTOR levels. miR-99 represses the expression of mTOR by directly binding to 3′UTR of its mRNA. miR-150-mediated silencing of mTOR was observed only when co-expressed with miR-99a, suggesting a functional synergy between miRNAs (Warth et al., 2015).

To understand the miRNA function in Treg development, Yogesh Singh and his colleagues searched for important miRNAs and their relevant target genes. miR-15b/16, miR-24, and miR-29a were found to impact induced Tregs (iTregs) *in vitro via* the mTOR signaling pathway (Singh et al., 2015; Singh et al., 2015). miR-181 also played an important role in T-cell development, while the mechanisms remain only partially explored (Li et al., 2007; Neilson et al., 2007). miR-181 is a family composed of six miRNAs, miR-181a/b-1, miR-181a/b-2, and miR-181c/d. Investigations suggested that miR-181a overexpression augmented T-cell receptor (TCR) signaling strength by targeting several protein tyrosine phosphatases, which displayed negative regulatory functions in TCR signaling (Li et al., 2007). In the absence of miR-181a/b-1, TCR signals are insufficient to produce Foxp3^+^precursors. Nevertheless, it has been suggested that miR-181 acts as an important regulator of T-cell development (Kim et al., 2021).

### miRNAs involved in Th17/Treg homeostasis by regulating Treg induction and stability

**FIGURE 3:**
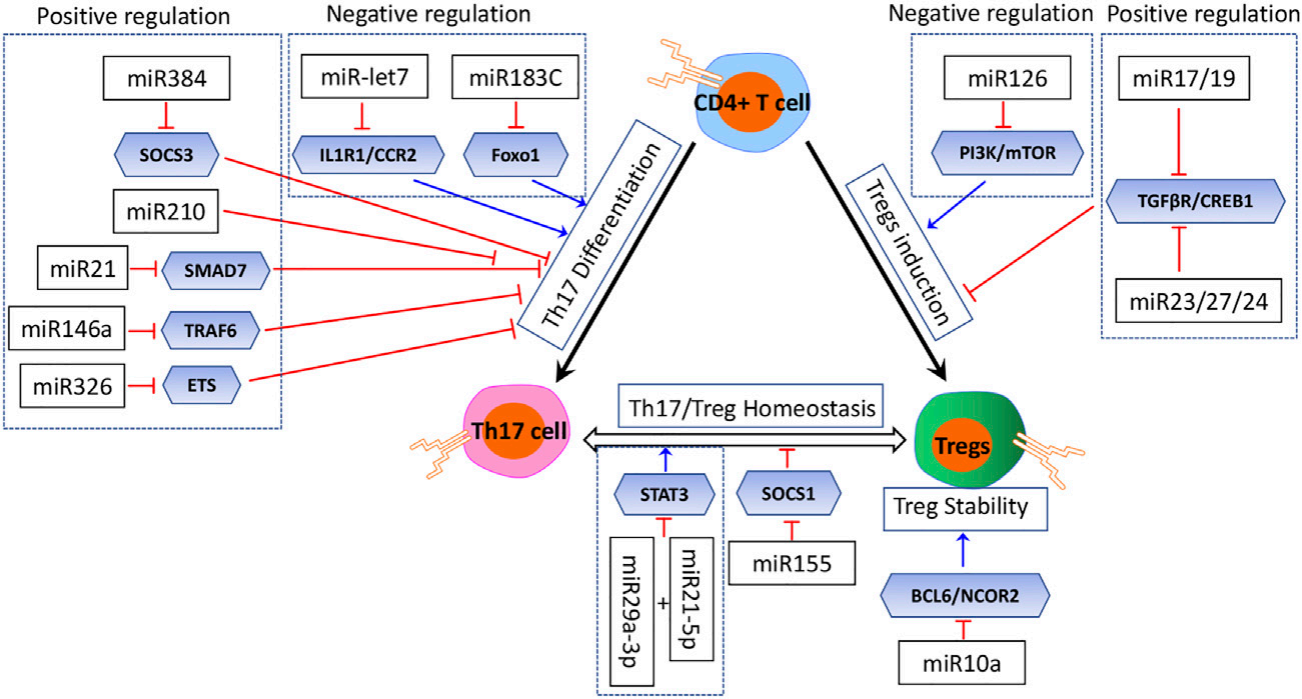
miRNAs in Treg induction and Th17 differentiation.

Treg induction *in vivo* is most likely controlled by multiple miRNAs, which might act in concert or in an isolated manner (Figure 3) (Curotto de Lafaille and Lafaille, 2009). The *in vitro* Treg induction in the presence of TGFβ is also dependent on proper regulation by miRNAs (Cobb et al., 2006; Chong et al., 2008). Multiple studies had identified miRNAs with both positive and negative regulatory effects on Treg induction (Warth et al., 2015). The PI3K/Akt/mTOR pathway with several miRNAs forms a network which positively regulates Treg induction cooperatively. Qin reported that miR-126 could upregulate Treg induction by inhibiting the activity of PI3K/Akt/mTOR, while in the absence of miR126, the activity of the PI3K/Akt/mTOR pathway was enhanced, and Foxp3 expression and Treg induction were decreased (Qin et al., 2013). On the other hand, several miRNAs have a negative effect on Treg induction (Warth et al., 2015). For example, miR17 and miR19, members of the miR17∼92 cluster, function as negative regulators of Treg induction while being dispensable for thymic Treg development (Jiang et al., 2011). miR17 directly targets TGFβ-receptor II and the cAMP-responsive element-binding protein 1 (CREB1), both involved in proper Treg induction. The TGFβ signaling pathway is also a target of the miR23-miR27-miR24 cluster, and an overexpression of this cluster impairs Treg induction (Cho et al., 2016). miR-31 was also reported to negatively regulate the Treg induction by inhibiting retinoic acid-inducible protein 3, which was validated in an experimental autoimmune encephalomyelitis (EAE) model in mice (Zhang et al., 2015).

The essential role of miRNAs in Treg stability had also been highlighted by the Treg-specific ablation in mouse models; these miRNAs deficiency resulted in disturbed Treg stability (Cobb et al., 2006; Liston et al., 2008; Zhou et al., 2008). As described before, miR-155 is highly expressed in Tregs (Naqvi et al., 2021). miR155 deficiency not only resulted in a reduced frequency of Tregs and impaired Treg development but also resulted in disturbed Treg stability by targeting SOCS1, a negative regulator of STAT5 signaling which determines the responsiveness to IL2 (Kohlhaas et al., 2009; Lu et al., 2009; Yao et al., 2012). miR-10a is a Treg-specific miRNA and had been identified to stabilize the Treg-specific gene expression by targeting effector T-cell genes *Bcl6* and *Ncor*2 (Jeker et al., 2012; Takahashi et al., 2012). Also, miR-10a controls the stability of FoxP3 expression in peripherally derived and *in vitro*-induced Tregs (Takahashi et al., 2012). Takahashi showed that miR-10a was induced synergistically with TGF-β and retinoic acid in conventional CD4+ T cells and was required for the stability of FoxP3 expression rather than the function of Tregs (Takahashi et al., 2012). However, Jeker et al. (2012) suggested that miR-10a may be indispensable for Treg stabilization and function. To summarize, miR-155 had been identified as one of the most predominant miRNAs in Treg induction, while it is still controversial that whether miR-10a is involved in Treg induction.

### miRNAs involved in Th17/Treg homeostasis by regulating Th17 differentiation

Th17 differentiation is also regulated tightly by miRNAs (Figure 3) (Ghoreschi et al., 2010; Lee et al., 2012). Naïve CD4^+^ T cells are differentiated to the Th17 phenotype with the treatment of TGF-β, IL-6, and IL-1 (Ivanov et al., 2006; Yang et al., 2008; Korn et al., 2009). One potential mechanism for the fine tuning of this balance is miRNAs; however, the roles of miRNAs that intrinsically control Th17 differentiation remain elusive. Based on the current investigation, several miRNAs were reported to play an inhibitory role. Ichiyama et al. (2016) reported five upregulated miRNAs (miR-183, miR-96, miR-182, miR-10b, and miR-351) in Th17 cells compared with the other Th subsets by RNA sequencing, and the upregulated miR-183C cluster (miR-183, miR-96, and miR-182) with its direct target (Foxo1) could suppress Th17 differentiation by RORγt. Additionally, a study by Angelou demonstrated that let-7 miRNAs negatively regulated Th17 proliferation and differentiation *via* directly targeting the cytokine receptors Il1r1, Il23r, Ccr2, and Ccr5 (Angelou et al., 2019). Another study by Wu et al., (2018) reported that the overexpression of miR-210 in psoriasis had a negative effect on Th17 differentiation.

However, they also had some miRNAs which played an inducing role in Th17 differentiation (Figure 3). Qu and his colleagues revealed that miR-384 was critically involved in EAE. They had also identified that miR-384-favored naïve T cells toward Th17 differentiation *in vitro* by targeting the suppressor of cytokine signaling 3 (SOCS3) (Liu et al., 2015; Qu et al., 2012) Hosseini et al. (2016) showed that miR-223 could modulate chemokine signaling to promote Th17 differentiation and suppress Treg differentiation, highlighting its potential role in maintaining the Treg/Th17 balance. A study by Howard L.Weiner had characterized that miR-21 was involved in a T-cell–intrinsic miRNA pathway that enhanced TGF-β signaling and then promoted Th17 differentiation by targeting SMAD-7, a negative regulator of TGF-β signaling (Murugaiyan et al., 2015). Furthermore, the essential role of miR-21 was highlighted in a mouse model of EAE. miR-21 expression was elevated in Th17 cells, and the mice lacking miR-21 had a defect in Th17 differentiation and were resistant to EAE; except that, miR-146a was demonstrated to be involved in Th17 differentiation *via* targeting TRAF6 (Li et al., 2017; Li et al., 2019). miR-326 also impacts Th17 differentiation by targeting the ETS transcription factor family (Du et al., 2009). Additionally, J. Zhou identified that miR-29a-3p and miR-21-5p had a synergistic effect on STAT3 inhibition and regulated Treg/Th17 cells, therefore inducing an imbalance (Zhou et al., 2018).

Furthermore, Kamran Ghaedi developed a network of autoimmune-deregulated miRNAs in Th17 differentiation. Several miRNAs and their downstream regulators involved in Th17 differentiation have been discovered by using the integrative miRWalk database. They nominated several miRNAs which probably may have a strong possibility for inducing an inhibitory role in Th17 differentiation, respectively (positive miRNAs include miR-27b, miR-27a, miR-30c, miR-1, miR-141, and miR-20b; negative miRNAs include miR-93, miR-20a, miR-152, miR-21, and miR-106a) (Honardoost et al., 2015).

### miRNAs involved in Th17/Treg homeostasis by regulating Treg function and cytokine secretion

Evidence suggests that miRNAs played a crucial role in cytokine secretion and Treg function in autoimmune diseases.(Valencia et al., 2006; Bayry et al., 2007; Nadkarni et al., 2007; Buckner, 2010). Here, we summarize these critical miRNAs involved in Treg function. It had been elucidated that miR-155 and miR-146a are two essential regulators in regulating Treg cell function in many autoimmune diseases. miR-155 was required for the release of cytokine production such as IL-2 and IFN-γ (Vigorito et al., 2007; Lu et al., 2009). Moreover, its mimics induced cytokine production, while the miR-155 knockout attenuated cytokine release in response to antigen stimulation (Rodriguez et al., 2007; Thai et al., 2007; Sharma et al., 2016). miR-146a is highly expressed in Tregs and exerts the orchestration of immunosuppressive signaling events in T effector cells and Tregs (Lu et al., 2010). miR-146a restrains IFN-γ mediated pathogenic Th1 responses. In addition, it maintains the Treg function by regulating pro-inflammatory cytokines, such as TNF-a, IFN-γ, and IL-17, and its target is STAT1, which is a key transcription factor required for T-cell response. Its critical role had been verified that miR-146a deficiency resulted in an impaired Treg function in the mouse model (Lu et al., 2010). In addition, the miR17-92 cluster, which includes miR-17, miR-18a, miR-19a, miR-20a, miR-19b, and miR-92, is reported to play an important role in Treg function under the inflammatory status by preserving antigen-specific Tregs and regulating immunosuppressive IL-10 secretion in Tregs (Jiang et al., 2011). Yang et al. (2016) reported that miR-17 could regulate the suppressive function of Tregs and effector T cells *via* inhibiting Eos and other transcriptional co-regulators in an IL-6-enriched environment. However, it is not essential for Treg regulation under non-inflammatory status.

High cAMP concentration maintenance is essential for the suppressive function of Tregs. Graham M and his colleagues demonstrated that miR-142-5p acts as an immune regulator of intracellular cAMP, thereby controlling Treg suppressive function. Deletion of miR-142-5p in Treg would result in multisystem autoimmunity (Anandagoda et al., 2019). On the other hand, miR-142-3p was also demonstrated to regulate Tregs’ suppressive function by inhibiting the expression of adenylyl cyclase 9, which is responsible for generating cAMP (Huang et al., 2009). The miR-106b-25 cluster, which includes mir-25, mir-106b, and mir-93, is involved in TGF-beta production which is essential for Treg maturation (Ha, 2011; Liu et al., 2020). miR-15a/16 was shown to regulate Tregs’ suppressive function by inhibiting CTLA-4 expression (Liu et al., 2014). miRNA let-7d was shown to enhance Tregs’ suppressive function by inhibiting Th1 proliferation and cytokine secretion (Okoye et al., 2014; Geng et al., 2020).

### miRNAs involved in uveitis pathogenesis by regulating Treg/Th17 homeostasis in animals

As summarized previously, accumulating evidence had revealed the critical significance of miRNAs in the Treg/Th17 balance for autoimmune diseases, which share similar pathological characteristics with autoimmune-mediated eye diseases, including autoimmune uveitis. However, it also had a personalized pathology because of the fact that the eyeball is an immune-privileged organ and has adapted several negative regulators to suppress inflammation by mediating Treg cells (Benhar et al., 2012; Taylor and Ng, 2018). Epigenetic mechanisms in autoimmune uveitis had been investigated in several studies; however, it has not been fully explored at present. Here, we reviewed miRNAs involved in uveitis pathogenesis by regulating Th17 or Treg cells.

We included eight animal studies about experimental autoimmune uveitis (EAU) which is the classic animal model of uveitis (Caspi et al., 2008) (Bansal et al., 2015). As is summarized in Table 1, several miRNAs had been demonstrated to be regulated and play a vital role in EAU. Among them, miR-223-3p has been reported as an important miRNA in the Tregs/Th17 homeostasis *via* several different mechanisms. A study by MX Zhou found that upregulated miR-223-3p regulated Th1 and Th17 differentiations by the transcription factor Rbpj. Another study by Yankai Wei found that miR-223-3p promoted autoreactive Th17 cell responses by inhibiting the FOXO3 expression. In addition, T Watanabe and his colleague found that the overexpression of miR-223-3p was closely associated with the elevation of L-1β/MCP-1. In the EAU animal model, miR-155 and miR-146a had also been verified as two essential regulators in Tregs/Th17 homeostasis. Escobar et al. (2014) demonstrated that miR-155 and STAT3/miR-155 axes contributed to EAU development by modulating the Th17 cell differentiation. miR-146a was found to be upregulated in EAU retina; moreover, its overexpression was closely associated with the inflammation score of EAU by regulating IL-1β/MCP-1 and IL-10 and IL-17 (Watanabe et al., 2016). In EAU, miR-30b-5p was reported to be involved in disease development by targeting IL-10 and TLR4 in T cells (Sun et al., 2018). It was well demonstrated that miR-142-5p could control Tregs’ suppressive function by regulating intracellular cAMP (Talebi et al., 2017; Anandagoda et al., 2019), and miR-21 could promote Th17 differentiation by regulating TGF-β signaling. In EAU, both miR-142-5p and miR-21 were found to be overexpressed in ocular tissues and corresponding with the dynamic expression of IL-17, indicating the involvement of miR-142-5p and miR-21 in the development of EAU by regulating Treg/Th17 hemostasis (Murugaiyan et al., 2015; Watanabe et al., 2016; Anandagoda et al., 2019). miR-181a, which could regulate T-cell and Treg differentiation by targeting TCR signaling, was observed to be downregulated in the rat model of EAU and was corresponding with the score of EAU (Li et al., 2007; Watanabe et al., 2016). miR-30b-5p had been reported as T-cell-associated miRNAs; however, its role in Treg/Th17 hemostasis had not been clearly elucidated. A study by YY Sun reported that downregulated miR-30b-5p could regulate the levels of IL-10- and TLR4-positive cells (Sun et al., 2018; Torri et al., 2017). Collectively, these observations indicate that miRNAs are vital regulators of EAU development by regulating the Treg/Th17 balance.

**TABLE 1:** miRNAs involved in uveitis pathogenesis by regulating Treg/Th17 homeostasis in animals.

Author	Species	Type of uveitis	miRNA	Potential role
MX Zhou et al. (2018)	Rats	EAU^a^	miR-223-3p (↑)	Regulating the transcription factor Rbpj on the differentiation of Th1 and Th17 cells
Shi et al. (2019)	Mice	EAU	miR-21-5p (↑)	Regulating the Th17/Treg balance *via* binding to the 3′-UTR of IL-10
Yankai Wei et al. (2020)	Mice	EAU	miR-223-3p (↑)	Promotes autoreactive Th17 cell responses by inhibiting FOXO3 expression
Escobar et al. (2013)	Mice	EAU	miR-155	Promote the expansion of pathogenic Th17 cells with STAT3
Ishida et al. (2011)	Mice	EAU	miR-142-5p and miR-21 (↑); miRNA-182 (↓)	Regulating Th17 development by affecting IL-17
Yuanyuan Sun et al. (2018)	Rats	EAU	miR-30b-5p (↓)	Regulating the level of IL-10- and TLR4-positive cells
T Watanabe et al. (2016)	Rats	EAU	miR-223 and miR-146a (↑); miRNA-181a (↓)	Associated with the elevation of IL-1β/MCP-1
Hsu et al. (2017)	Rats	EAU	miR-146a (↑)	Reduce inflammation by downregulating IL-1*β*, IL-6, IL-12, and IFN-*γ* and upregulating IL-10 and IL-17

a Experimental autoimmune uveitis (EAU); (↑) upregulated; (↓) downregulated.

Furthermore, differentially expressed miRNAs had been identified between diseased animal models and healthy control. Guo et al. had reported 36 upregulated miRNAs and 31 downregulated miRNAs in peripheral blood lymphocytes from EAU; moreover, these candidate miRNAs were closely associated with immune signaling and contributed to EAU development (Ishida et al., 2011; Watanabe et al., 2016; Wei et al., 2020). Hsu et al. (2015) had reported three upregulated miRNAs (miR- 9-3p, miR-182-5p, and miR-183-5p) and four downregulated miRNAs (miR-146a-5p, miR-155-5p, miR-147b, and miR-223-3p) in the retina from EAAU (Hsu et al., 2015).

### miRNAs involved in uveitis pathogenesis by regulating Tregs/Th17 in humans

Nine studies on uveitis patients were included, and the critical implications of miRNAs are summarized in Table 2. The types of uveitis are mainly Behçet’s disease (BD) and Vogt-Koyanagi-Harada (VKH) syndrome, which are systematic autoimmune diseases with eye involvement (Woo et al., 2016; Wei et al., 2020; Vega-Tapia et al., 2021). Extra ophthalmic involvement usually concerns the skin, central nervous system, gastrointestinal, and mucocutaneous disorders (Paovic et al., 2013; Baltmr et al., 2016).

**TABLE 2:** miRNAs involved in uveitis pathobiology by regulating Treg/Th17 in humans.

Author	Species	Type of uveitis	miRNA	Potential role
G Jadideslam et al. (2019)	Humans	Behçet’s disease	miR-326 (↑); miR-21 and miR-146b (↓)	Used as a biomarker for the prediction of uveitis and severe eye involvement
Rui Chang et al. (2018)	Humans	VKH^a^ syndrome	miR-20a-5p (↓)	Suppressing the production of IL-17 by targeting OSM and CCL1 production in CD4^+^ T cells
Sousan Kolahi et al. (2018)	Humans	Behçet’s disease	miR-155 and miR-146a (↑)	Associated with the upregulation of TNF-α and downregulation of CTLA-4 genes
Majid Ahmadi et al. (2019)	Humans	Behçet’s disease	miR-25, miR-106b, miR326, and miR93 (↑); miR-146a, and miR-155 (↓)	Associate with the Th17/Treg frequency and act as a prognostic biomarker
Yu et al. (2014)	Humans	Behçet’s disease and VKH	miR-182 (↑)	As regulatory factors for Treg cell development and function
So Young Na et al. (2016)	Humans	Behçet’s disease	miR-155 (↑)	Regulating the Th17 immune response by targeting Ets-1
Min Yeong Woo et al. (2016)	Humans	Behçet’s disease	miR-3591-3p (↑); miR-638 and miR-4488 (↓)	Increasing IL-6 mRNA levels in Th-1 cells in response to LPS stimulation
Qingyun Zhou et al. (2014)	Humans	VKH syndrome	miR-146a (↓)	Strong association with IL-17, TNF-α, and IL-1β production
Qingyun Zhou et al. (2012)	Humans	Behçet’s disease	miR-155 (↓)	Inhibiting the production of IL-6 and IL-1β, promoting the expression of IL-10, and inhibiting intracellular IL-17 expression in allogeneic CD4^+^ T cells by targeting TAB2

a Vogt–Koyanagi–Harada (VKH) syndrome.

Among these studies on BD, we found that miR-155 and miR-146a were significantly differentially expressed in the PBMCs; however, the result was controversial and its epigenic mechanism is still elusive (Kolahi et al., 2018; Woo et al., 2016; Zhou et al., 2012). Zhou et al. (2012) found that downregulated miR-155 inhibited the Th17 responses by targeting TGF-beta-activated kinase 1 binding protein 2 (TAB2), while Na et al. (2016) reported that miR-155 was upregulated and promoted Th17 responses *via* the suppression of E26 transformation- specific-1 (Ets-1) in BD. Kolahi et al. (2018) reported that upregulated miR-155 was associated with TNF-α and CTLA-4D. Another vital miRNA is miR-146a; its role in BD patient is also unelucidated and need to be further explored. Jadideslam et al. (2019) reported that the miR-146a expression had no significant difference between BD patients and healthy control. However, a study had identified that there is a strong correlation between upregulated miR-146a and TNF-α/CTLA-4 (Kolahi et al., 2018), whereas another study had identified that downregulated miR-146a was associated with the Th17/Treg frequency and could act as a prognostic biomarker (Ahmadi et al., 2019). In addition, miR-326, miR-21, miR-146b, miR-25, and miR-106b had been revealed to be differentially expressed in PBMCs from BD patients and were suggested to be biomarkers for predicting uveitis (Woo et al., 2016; Ahmadi et al., 2019; Jadideslam et al., 2019; Wei et al., 2020).

In addition to BD, VKH is also one of the main autoimmune uveitis with severe vision loss. Studies showed that VKH patients had a lower expression of miR-20a in CD4 + T cells, and upregulated miR-20a negatively regulated IL-17 expression *via* regulating OSM and CCL1 (Chang et al., 2018). Moreover, an analysis based on miRNA–mRNA interactions found that miR-20a may suppress Th17 differentiation through the targeting of several regulators (Honardoost et al., 2015). These findings highlighted the involvement of miR-20a in impaired Treg/Th17 balance during VKH. Several studies had indicated that differentially expressed miR-146a was linked to VKH closely. Evidence showed that miR-146a had a strong association with IL-17, TNF-α, and IL-1β production (Zhou et al., 2014). However, the exact mechanism of the miR-146a gene involved in VKH is still unknown. Additionally, abnormal encoding gene copy numbers of miRNAs (miR-23a, miR-301a, miR-182, and let-7g-3p) have been revealed to have a strong link with disease development (Vega-Tapia et al., 2021).

## Conclusion

Impaired Treg/Th17 homeostasis can impact immune tolerance and trigger inflammation that can eventually result in autoimmune diseases’ development. Preserving Treg/Th17 balance is a pivotal treatment strategy. Given the importance of miRNAs for the proper function of the immune system, a number of studies have investigated their epigenic mechanisms on Treg/Th17 hemostasis and their potential role in disease pathogenesis. Collectively, differentially expressed miRNAs were involved in Th17/Treg homeostasis by regulating Treg differentiation and development, Treg induction, Treg stability, Th17 differentiation and Treg function, and cytokine secretion. Considering the similar immunological characteristics in uveitis, we reviewed and summarized miRNAs and their molecular targets in uveitis for further investigation. Several miRNAs and their potential role in regulating Treg/Th17 balance have been identified in animal studies and human studies, respectively. Overall, establishing how miRNAs contribute to Treg/Th17 homeostasis will help define the epigenetic regulation in uveitis and other autoimmune diseases and open new avenues for treatment.

## Data Availability

The raw data supporting the conclusion of this article will be made available by the authors, without undue reservation.
